# Characterizing the cellular attachment receptor for Langat virus

**DOI:** 10.1371/journal.pone.0217359

**Published:** 2019-06-04

**Authors:** Raquel Rodrigues, Katarina Danskog, Anna K. Överby, Niklas Arnberg

**Affiliations:** Virology, Department of Clinical Microbiology, Umeå University, Umeå, Sweden; University of Minnesota, UNITED STATES

## Abstract

Tick-borne encephalitis infections have increased the last 30 years. The mortality associated to this viral infection is 0.5 to 30% with a risk of permanent neurological sequelae, however, no therapeutic is currently available. The first steps of virus-cell interaction, such as attachment and entry, are of importance to understand pathogenesis and tropism. Several molecules have been shown to interact with tick-borne encephalitis virus (TBEV) at the plasma membrane surface, yet, no studies have proven that these are specific entry receptors. In this study, we set out to characterize the cellular attachment receptor(s) for TBEV using the naturally attenuated member of the TBEV complex, Langat virus (LGTV), as a model. Inhibiting or cleaving different molecules from the surface of A549 cells, combined with inhibition assays using peptide extracts from high LGTV binding cells, revealed that LGTV attachment to host cells is dependent on plasma membrane proteins, but not on glycans or glycolipids, and suggested that LGTV might use different cellular attachment factors on different cell types. Based on this, we developed a transcriptomic approach to generate a list of candidate attachment and entry receptors. Our findings shed light on the first step of the flavivirus life-cycle and provide candidate receptors that might serve as a starting point for future functional studies to identify the specific attachment and/or entry receptor for LGTV and TBEV.

## Introduction

Tick-borne encephalitis virus (TBEV) is an important, emerging human pathogen and the causative agent of the most important arbovirus-caused disease in Europe and Russia [1,2]. It belongs to the tick-borne virus complex of the *Flaviviridae* family and *Flavivirus* genus, which also includes Langat, Kyasanur forest disease, Louping ill, Negishi, Omsk hemorrhagic fever and Powassan viruses [3], and are closely related to other arthropod-borne flaviviruses, including dengue, West Nile, yellow fever and Japanese encephalitis viruses [4]. Currently, no specific therapeutic exists for TBEV, however, there are several vaccines available as a preventive measure: two european (FSME-Immun/TicoVax, Pfizer Pharma and Encepur, Chiron Behring) and two russian, (TBE Moscow, Chumakov Institute and Encevir, Microgen) [5]. Despite this, each year 6000 to 8000 clinical cases are reported in Russia and 3000 to 4000 cases in other parts of Europe [6–9]. The symptoms of TBEV infection in humans, ranges from mild to severe forms of meningitis, encephalitis, and haemorrhagic fever [8] with a risk of permanent neurological sequelae of around 40% [10]. The mortality rate ranges from 0,5 to 30%, depending on the subtype. However, the mechanisms underlying this difference in pathogenicity are not completely understood [8].

The virion is an enveloped particle containing 11-kb of single-stranded, positive sense RNA which encodes ten viral proteins, of which three are structural proteins: capsid (C), membrane (M), and envelope (E) protein. The outer surface of the virus is covered by dimers of the E protein, which provides the virus with its antigenic properties [11] and mediates binding to and entry into the host cell [12,13]. The life cycle of flaviviruses begins with the attachment of the E protein to a host cell receptor, which subsequently initiates receptor-mediated endocytosis. A number of cellular receptors have been suggested for several flaviviruses [14]. For TBEV, studies have suggested three cell surface receptors: glycosaminoglycan heparan sulfate (HS) [15], human laminin*-*binding protein (LBP) [16] and human integrin αVβ3 [17]. However, these studies have not differentiated between TBEV attachment factors and entry receptors.

In this study, we sought to characterize the nature of the cellular attachment receptors for TBEV and to generate a list of candidate receptors. For this, we used Langat virus (LGTV), the naturally attenuated form of TBEV, as a model. Our data indicated that the attachment receptor(s) for LGTV are of protein nature. Moreover, the list of candidate receptors described here form a foundation for future studies on the specific attachment and entry receptors for LGTV and TBEV.

## Results

### Treatment with proteases impairs the binding of LGTV to A549 cells

**Fig 1:**
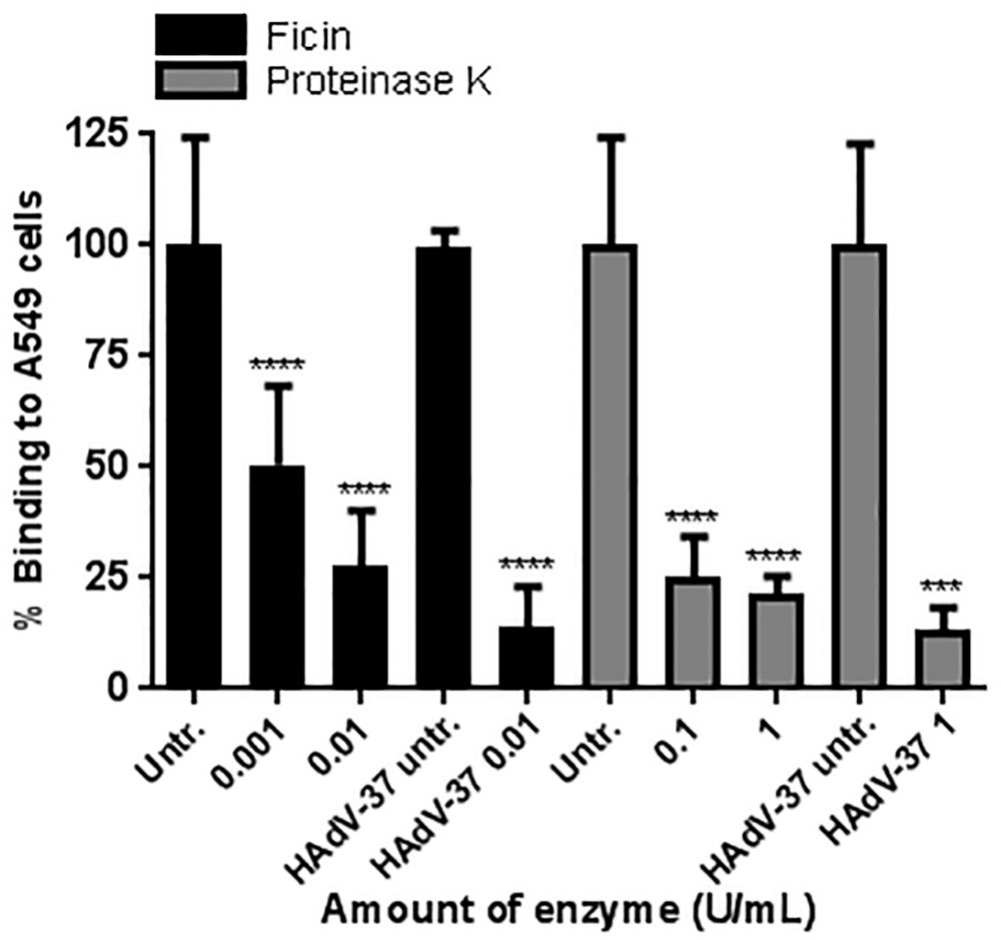
Protease treatment decreases binding of LGTV to A549 cells. To assess whether the cellular receptor for LGTV included a protein component, A549 cells were pre-treated with increasing amounts of the proteases Ficin and Proteinase K, prior to binding with LGTV. The bars indicate the percentage of cell-bound LGTV compared to untreated control. To demonstrate its effect, protease-treated cells were incubated with human adenovirus 37 (HAdV-37), shown to bind to proteins [21,22]. Results represent the mean of three independent experiments performed in duplicate. Error bars represent standard deviation. Statistical analysis was performed using one-way ANOVA with Dunnett’s multiple comparisons test for multiple comparisons and t test for single comparisons (***, P≤0.001; ****, P≤0.0001).

Cellular attachment receptors can be of protein, glycan or glycolipid nature. First, we investigated whether LGTV cellular attachment receptor(s) included protein components. For that, we treated A549 cells with increasing amounts of the proteases Ficin, a sulfhydryl protease which cleaves proteins at the carboxyl side of glycine, serine, threonine, methionine, lysine, arginine, tyrosine, alanine, asparagine and valine [18]; and Proteinase K, a relatively unspecific serine protease with a preference for aromatic and hydrophobic amino acids [19,20]. In this study, A549 cells were chosen due to its high susceptibility to binding and infection with LGTV. In our hands, after binding with an MOI of 10, the amount of cells with bound LGTV was approximately 70%. The viability of the cells after treatment was confirmed by trypan blue exclusion, showing that the treatments were not detrimental to the cell integrity. Treatment of A549 cells with 10 mU/mL of Ficin, prior to LGTV binding, inhibited the viral attachment by 75%, while 100 mU/mL of Proteinase K treatment resulted in 76% reduction. The effect of the proteases on A549 cells was demonstrated using human adenovirus 37 (HAdV-37), which was shown to bind to protein components of the cellular membrane [21,22]. As expected, pre-treatment of A549 cells with these proteases efficiently reduced (86 and 86.5% respectively) the binding of HAdV37 to A549 cells, confirming that the proteases were functional (Fig 1). This suggested that LGTV cellular attachment receptor(s) included one or more protein components.

### Glycans are not involved in LGTV attachment to A549 cells

We evaluated the involvement of glycan components in LGTV binding to host cells. For that, A549 cells were treated with compounds that cleave or inhibit *de novo* synthesis of HS, sialic acid (SA), *N*-linked glycans, *O*-linked glycans or glycolipids. For each of these compounds, the viability of the cells after the treatments was confirmed by trypan blue exclusion.

**Fig 2:**
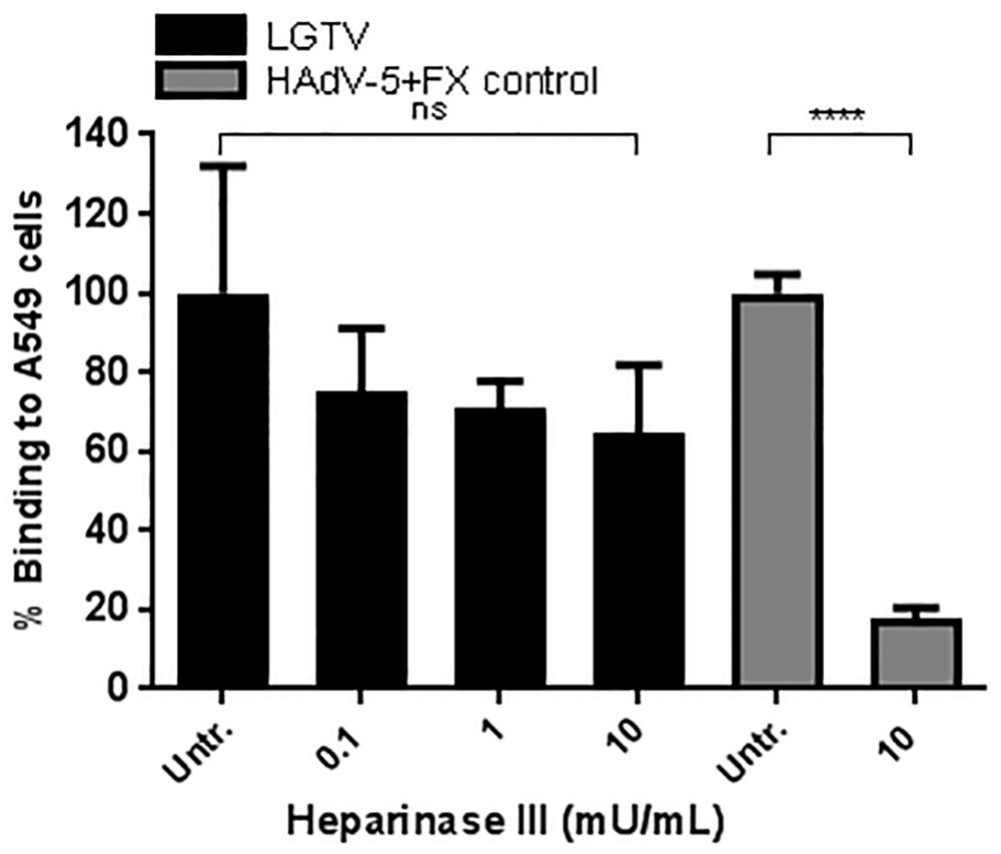
Heparan sulfate (HS) is not involved in LGTV binding to A549 cells. A549 cells were pre-incubated with increasing amounts of Heparinse III to cleave HS (black bars). To demonstrate its effect, Heparinase III-treated cells were incubated with pre-formed complexes of human adenovirus 5 (HAdV-5) virion:coagulation factor X (FX) (grey bars). Data in the figure represents means of three independent experiments performed in duplicate. Error bars represent standard deviation. Statistical analysis was performed using one-way ANOVA with Dunnett’s multiple comparisons test for multiple comparisons and t test for single comparisons (****, P ≤ 0.0001; ns, non-significant).

Previous studies have suggested HS to act as an attachment factor for several flavivirus including TBEV[15], however, data is lacking regarding HS involvement in LGTV binding. Therefore, A549 cells were treated with 10-fold dilutions of Heparinase III to remove HS from cell surface proteoglycans [23], before quantification of LGTV binding. No statistically significant reduction in binding was observed in comparison to the untreated cells (Fig 2), suggesting that HS is not important for LGTV binding to A549 cells. The effect of Heparinase III on A549 cells was demonstrated using pre-formed complexes of human adenovirus 5 (HAdV-5) virion:coagulation factor X (FX), which are known to bind to HS [24]. As expected, pre-treatment of A549 cells with 10U/mL of Heparinase III efficiently reduced (83%) the binding of HAdV-5:FX complexes to A549 cells, showing that Heparinase III was functional (Fig 2).

**Fig 3:**
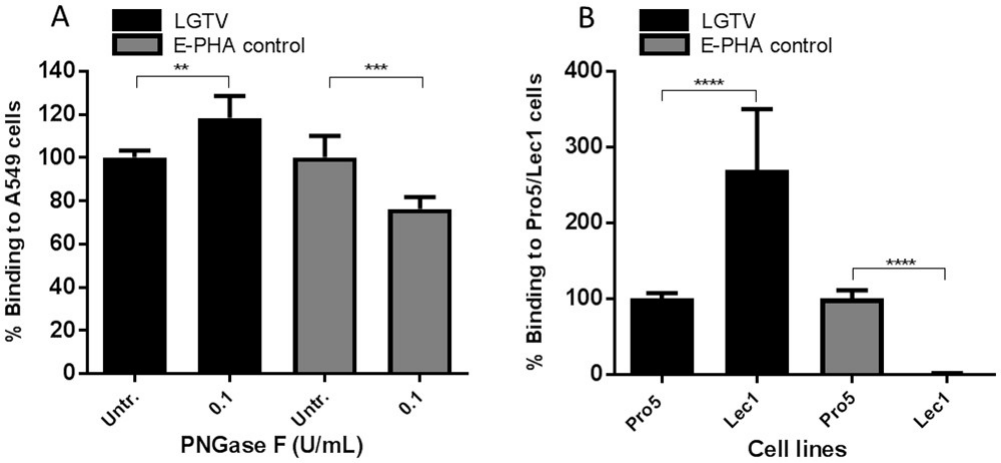
LGTV cellular receptor does not include *N*-linked glycans. (A) A549 cells were pre-incubated with 100 mU/mL of PNGase F (black bars). (B) The parental cell line Pro-5 and the *N*-glycan deficient cell line Lec1 were assessed for LGTV binding (black bars). (A and B) To demonstrate the reduction in *N*-linked glycan content, cells were incubated with the lectin *Phaseolus vulgaris* erythroagglutinin (E-PHA) (grey bars). Data in the figure represents means of three independent experiments performed in duplicate. Error bars represent standard deviation. Statistical analysis was performed using t test (**, P ≤ 0.01; ***, P ≤ 0.001; ****, P ≤ 0.0001).

Next, we investigated a potential involvement of *N*-linked and *O*-linked glycans in the attachment of LGTV to host cells. For that, we treated A549 cells with the Peptide *N-*Glycosidase F (PNGase F), an enzyme that is able to cleave *N-*linked glycans between an asparagine residue and the subsequent *N-*acetylglucosamine (GlcNAc) on the cell surface glycoproteins [25]. No reduction in LGTV binding to A549 cells was observed (a slight, but statistically significant, increase was observed) (Fig 3A). The efficiency of PNGase F treatment was confirmed by the decrease in binding of the lectin *Phaseolus vulgaris* erythroagglutinin (E-PHA), which is known to bind monosaccharides in the core of *N*-linked glycans [26] (Fig 3A). To confirm these results, we made use of the CHO-derived cell lines Pro-5 and Lec1, where Lec1 cell line is deficient in *N-*linked glycan production as compared to the parental Pro-5. Despite the fact that the amount of *N*-glycans on Lec1 cells was shown to be approximately 1% of the Pro-5 cells, no decrease in LGTV binding was found for Lec1 as compared to Pro-5 (an increase of 2.7-fold was observed) (Fig 3B), which is in accordance with the results described for PNGase F.

**Fig 4:**
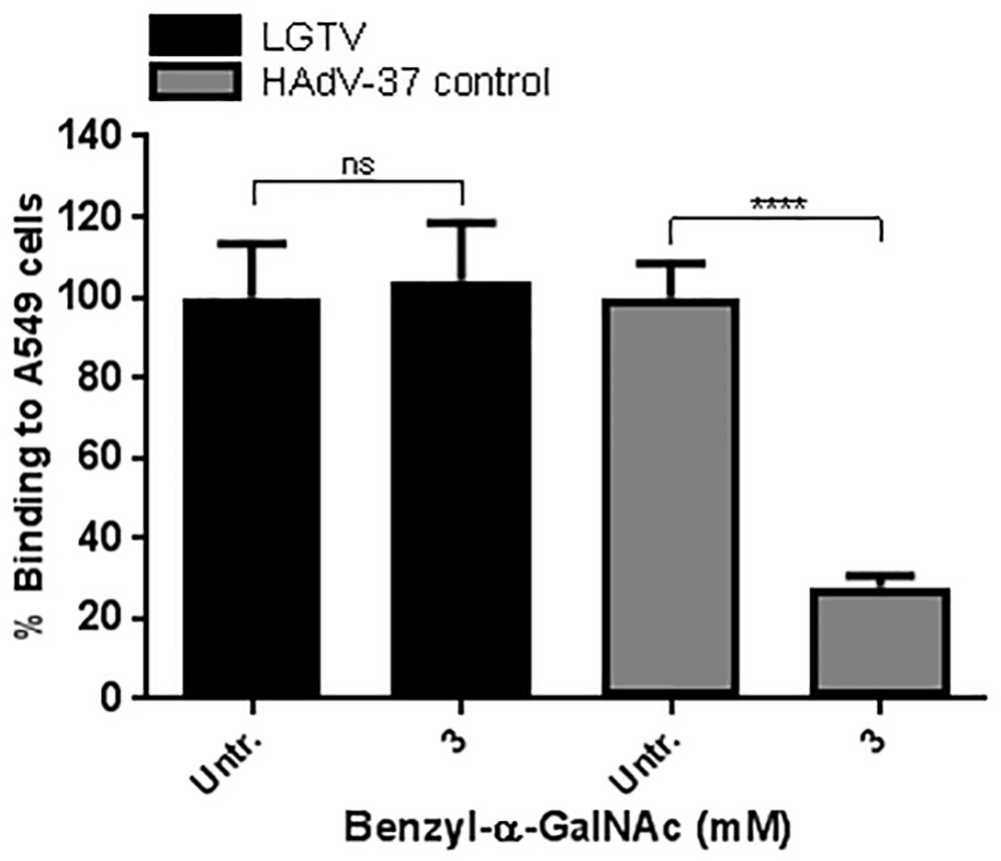
LGTV binding to A549 cells does not depend on *O*-linked glycans. A549 cells were treated with benzyl-α-GalNAc to inhibit *O*-glycosylation (black bars). As a control for this inhibition, HAdV-37 was used to bind to A549 cells (grey bars). Data represents means of three independent experiments performed in duplicate. Error bars represent standard deviation. Statistical analysis was performed using t test (**, P ≤ 0.01; ns, non-significant).

Benzyl-α-GalNAc inhibits *N*-acetyl-α-d-galactosaminyltransferase and thus blocks the first step of the *O*-linked glycosylation [27]. Here we used this molecule to assess the involvement of *O*-linked glycans in LGTV binding to A549 cells. Pre-treatment of A549 cells with Benzyl-α-GalNAc did not reduce LGTV binding (Fig 4), suggesting that *O*-linked glycans are not important for LGTV binding to A549 cells. The effect of benzyl-α-GalNAc on A549 cells was confirmed by using human adenovirus 37 (HAdV-37), which has been shown to bind to *O*-linked GD1a glycans on glycoproteins [22] (Fig 4). Together, this suggests that LGTV does not require *N*- or *O*-linked glycans for binding to A549 cells.

**Fig 5:**
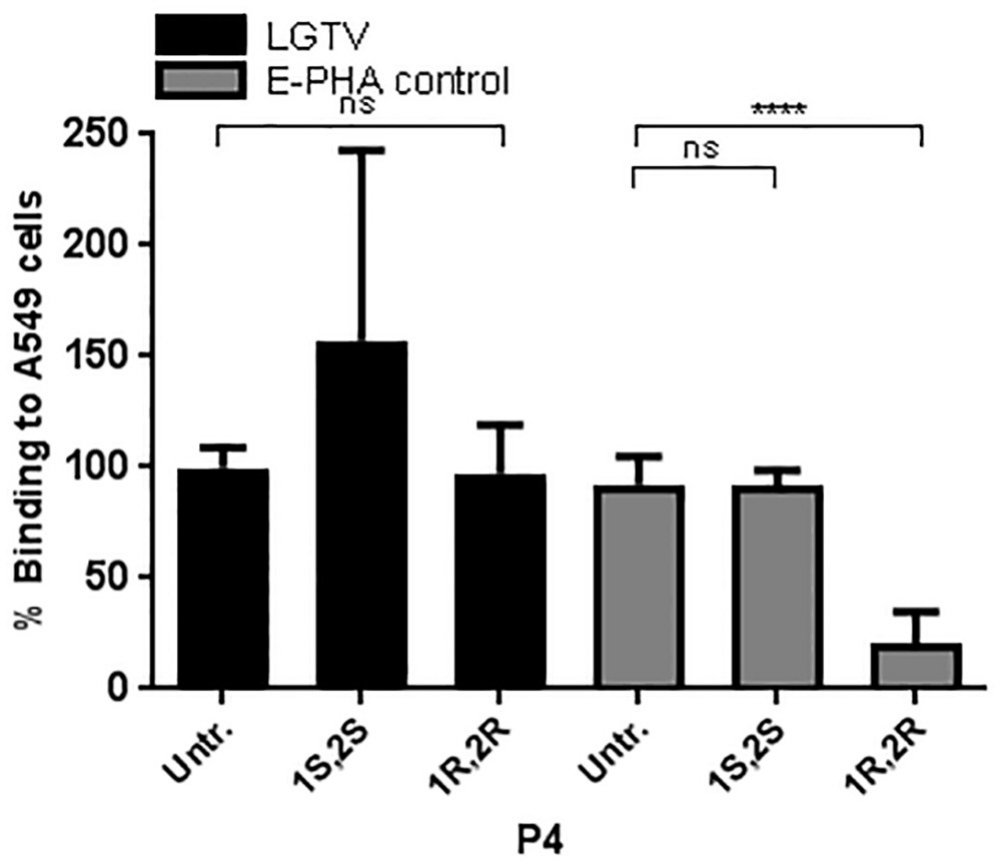
LGTV binding to A549 cells does not depend on glycolipids. A549 cells were pre-incubated with DL-threo-1-phenyl-2-palmitoylamino-3-pyrrolidino-1-propanol (P4), in its active (1*R*,2*R*) or inactive (1*S*,2*S*) form to inhibit glycolipid synthesis (black bars). To confirm its effect, P4-treated cells were incubated with FITC-conjugated cholera toxin B (CT-B) (grey bars). Data represents means of three independent experiments performed in duplicate. Error bars represent standard deviation. Statistical analysis was performed using one-way ANOVA with Dunnett’s multiple comparisons test for multiple comparisons and t test for single comparisons (****, P ≤ 0.0001; ns, non-significant).

Furthermore, the role of glycosphingolipids in the attachment of LGTV to A549 cells, was studied by treating A549 cells with (1*R*,2*R*)-1-phenyl-2-hexadecanoylamino-3-pyrrolidino-1-propanol (P4), a glycolipid biosynthesis inhibitor [28]. This treatment did not reduce LGTV binding as compared to the untreated control (Fig 5), suggesting that gangliosides are not involved in LGTV binding to A549 cells. The effect of P4 on A549 cells was confirmed by using the FITC-labeled, pentameric, ganglioside G_M1_-binding cholera toxin subunit B (CT-B) [29](Fig 5).

**Fig 6:**
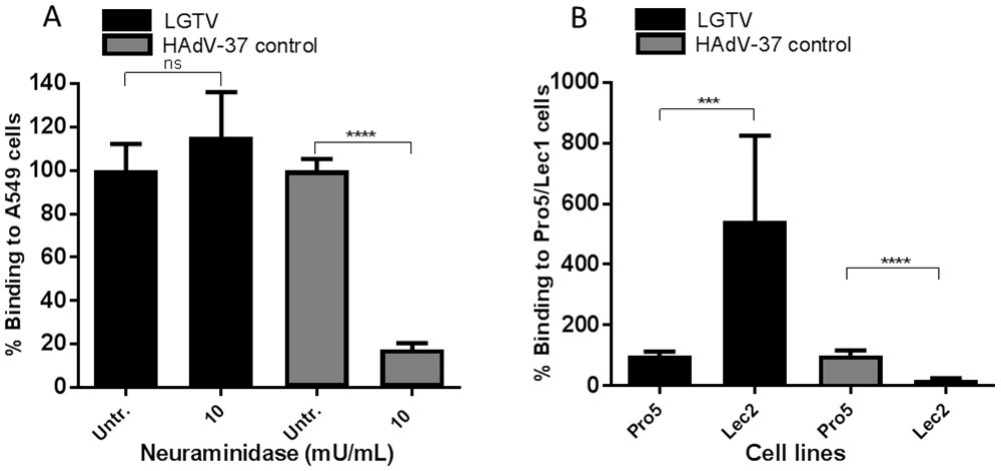
LGTV cellular receptor does not include sialic acid components. (A) A549 cells were pre-incubated with 10 mU/mL of *V*. *Choleare* neuraminidase (black bars). (B) The parental cell line Pro-5 and the sialic-acid deficient cell line Lec2 were assessed for LGTV binding (black bars). (A and B) To demonstrate the reduction in sialic acid content, cells were incubated with HAdV-37, known to bind to sialic acids (grey bars). Data in the figure represents means of three independent experiments performed in duplicate. Error bars represent standard deviation. Statistical analysis was performed using t test (***, P ≤ 0.001; ****, P ≤ 0.0001; ns, non-significant).

The mammalian central nervous system (CNS) has the highest concentration of SA [30]. Since LGTV and TBEV are neurotropic viruses, we investigated a possible role of SA as an attachment receptor for LGTV. Pre-treatment of cells with SA-cleaving neuraminidase [31] did not reduce the binding of LGTV to A549 cells as opposed to the decrease of the SA-binding control HAdV-37, at a concentration of 10mU/mL [32] (Fig 6A). This suggests that SA-containing molecules are not required for the binding of LGTV to A549 cells. To further investigate this assumption, we made use of the CHO-derived cell lines Pro-5 and Lec2, the latter being a mutant clone derived from the parental cell line Pro-5, with a 10-fold reduction in SA content. The results obtained after LGTV binding are consistent with those described for neuraminidase, as no decrease in binding was found (an increase of 5,4-fold was observed). As a control for the absence of SA of Lec2 as compared to Pro-5, we used HAdV-37 (Fig 6B). Overall, these results suggest that SA-containing glycans are, most likely, not required for the binding of LGTV to A549 cells.

### Correlation of LGTV binding with microarray gene expression pattern of NCI60 cell lines

Our results suggested that cell surface proteins are most likely involved in LGTV binding to A549 cells. Thus, we decided to undertake a transcriptomic approach to generate a list of receptor candidate proteins. For this, we evaluated the degree of LGTV binding to 60 cell lines of the NCI60 panel of human tumor cells, for which the gene transcript expression levels have been determined by multiple laboratories on multiple platforms [33–37]

**Fig 7:**
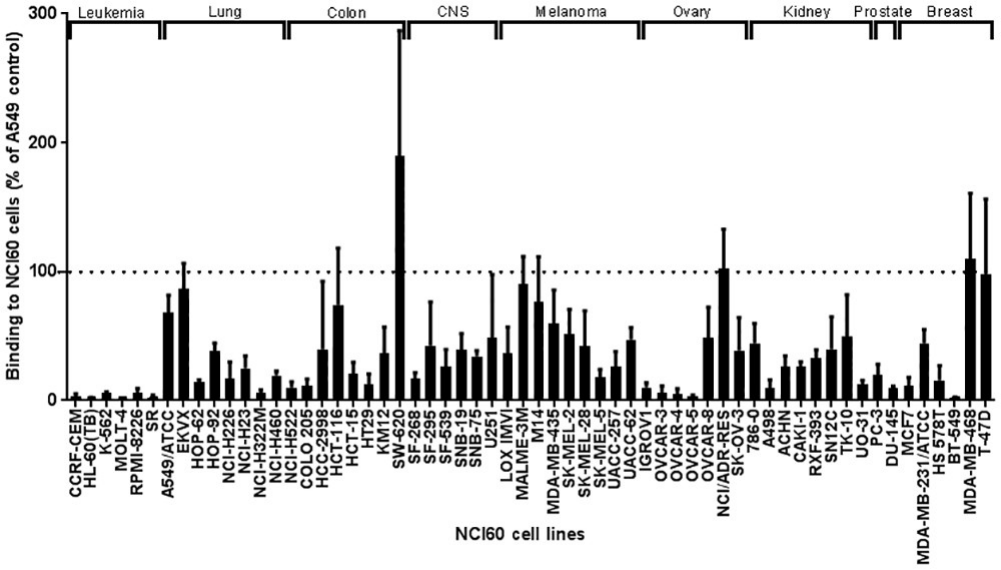
Binding of LGTV to 60 cell lines of the NCI60 panel. Each cell line of the NCI60 panel was assessed for the binding of LGTV. The results are expressed in percentage as compared to the control cell line A549, which was normalized to 100% (represented by the dotted line). Data in the figure represents the means of at least three independent experiments performed in duplicate. Error bars represent standard deviation.

After quantification of LGTV binding to each cell line, the values were normalized to the positive control (A549 cell line). The results are represented as a percentage of the control (Fig 7). Among the 60 cell lines, the highest binding was observed for the colon cancer cell line SW620, which had an average of twice as much binding as the A549 control. Apart from the leukemia and prostate cell lines, we observed LGTV attachment to all cell types, although the attachment levels varied within the same cell type or organ.

The binding values of LGTV to these cell lines were used to perform a correlation analysis with the known gene expression patterns of these cells [33–37] (RNA transcript data available online https://dtp.cancer.gov/databases_tools/molecular_target/default.htm). 10 out of the 60 cell lines were excluded from this analysis due to inconsistent results (HCC-2998, HCT-116, SF-295, U251, MDA-MB-435, SK-MEL-28, OVCAR-8, SK-OV-3, SN12C and TK10). The candidate receptors obtained by using this method are shown in S1 Table. The gene transcripts are ranked according to the correlation values. High correlation values correspond to genes that are highly expressed in cells for which higher binding values were determined. From this list, relevant candidates were selected according to the following criteria: highest correlation values, p value <.0001 and genes encoding cell-surface associated proteins. The selected candidates are highlighted in S1 Table: LGR6, the leucine-rich repeat containing G protein-coupled receptor 6; GPR155, the G protein-coupled receptor 155; KRTAP3-3, the keratin associated protein 3–3; MYH7B, myosin-7B; GFRa3, the glial cell line-derived neurotrophic factor receptor alpha-3; NKD1, naked-1 protein; SYT1, Synaptotagmin I; ASPSCR1, the tether containing UBX domain for GLUT4; RIMBP2, RIMS binding protein 2; TMEM176B, transmembrane protein 176B; TMEM176A, transmembrane protein 176A; KCNK10, potassium channel subfamily K member 10; PSD2, pleckstrin and sec7 domain containing 2; IGDCC3, the putative neuronal cell adhesion molecule and FZD10, Frizzled 10.

**Fig 8:**
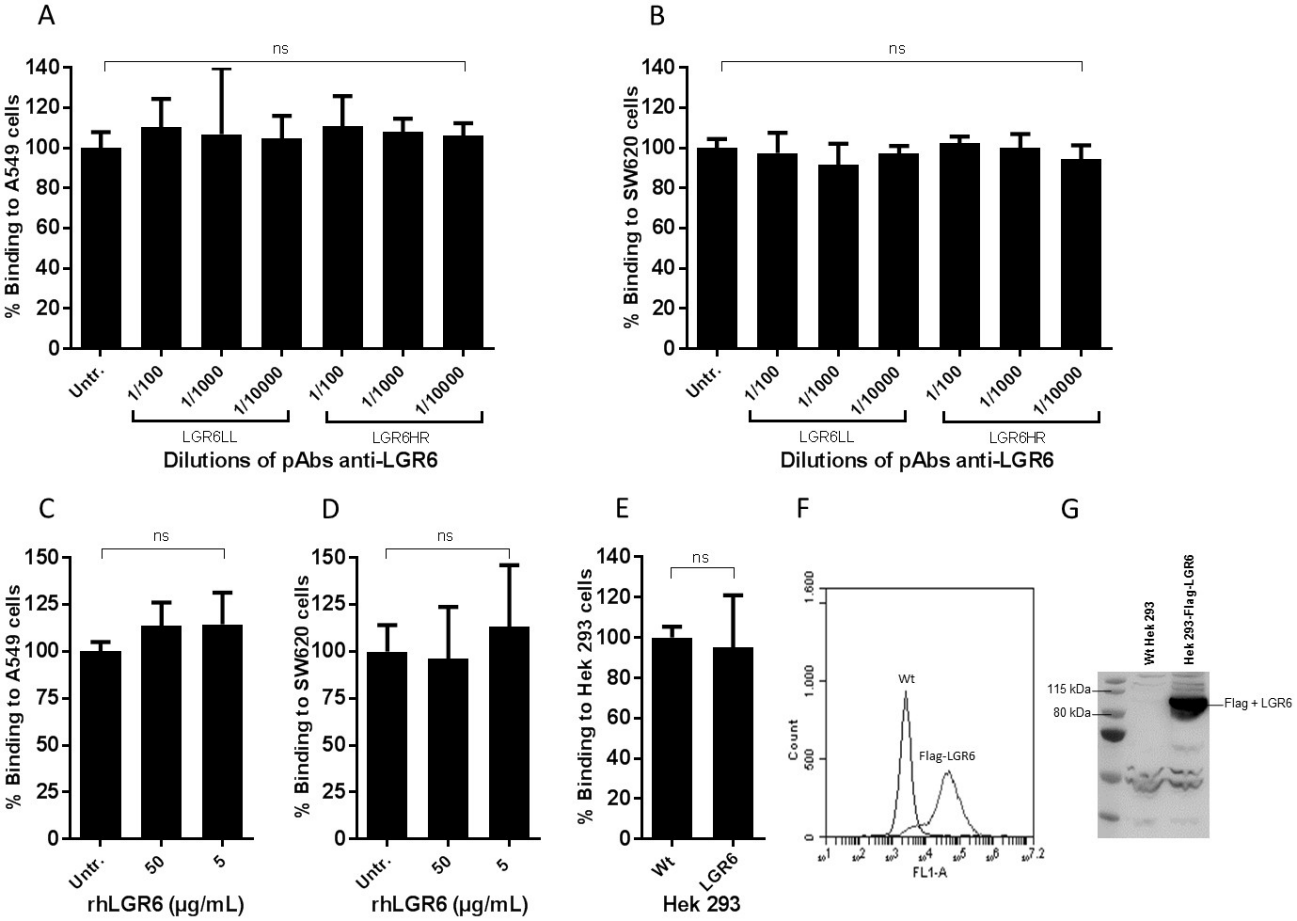
Inhibition or overexpression of LGR6 does not influence LGTV binding to A549 or SW620 cell lines. (A) A549 and (B) SW620 cells were pre-treated with 10-fold dilutions of two pAbs anti-LGR6: anti-LGR6 LL and HR. LGTV was pre-incubated with 50 or 5 μg/mL of rhLGR6, prior to binding with (C) A549 and (D) SW620 cells. (E) Hek293 cells were transfected with flag-LGR6 prior to binding to LGTV. Overexpression of flag-LGR6 was confirmed by flow cytometry (F) and western blotting (G) by using an anti-flag monoclonal antibody (mAb). The bars in the figures indicate the percentage of cell-bound LGTV compared to the untreated control. Data in the figure represents means of at least three independent experiments performed in duplicate, except for (C), for which two experiments were performed. Error bars represent standard deviation. Statistical analysis was performed using one-way ANOVA with Dunnett’s multiple comparisons test for multiple comparisons and t test for single comparisons (ns, non-significant).

From these selected candidates, LGR6 was the one with the highest correlation value. Thus, we sought to determine whether this protein could be the cellular binding partner for LGTV. For that, we i) pre-incubated A549 and SW620 cells with increasing amounts of polyclonal antibodies (pAbs) raised against the extracellular domain of LGR6 (Fig 8A and 8B), ii) pre-treated LGTV with recombinant human LGR6 in an attempt to inhibit LGTV binding (Fig 8C and 8D), and iii) overexpressed LGR6 in Hek293 cells (Fig 8E to 8G). The viability of the cells after the treatments was confirmed by the trypan blue exclusion test [38]. From these analyses, it is suggested that that LGR6 is not involved in LGTV binding to these cells.

### SW620 trypsinized membrane protein extract inhibits LGTV binding to SW620 cells but not to A549 cells

**Fig 9:**
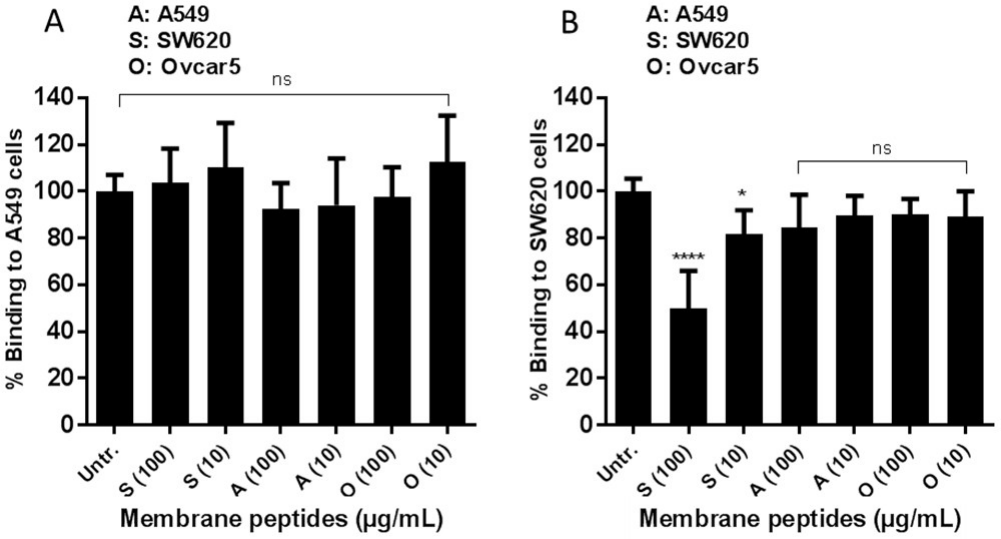
SW620 trypsinized peptides inhibit binding of LGTV to SW620 but not A549. (A) A549 cells and (B) SW620 cells were tested for the binding of LGTV after pre-incubation with 100 or 10 μg/mL of trypsinized membrane proteins from A, A549; S, SW620 and O, Ovcar5, a control cell line to which LGTV does not bind. The bars in the figures indicate the percentage of cell-bound LGTV compared to the control. Data in the figure represents means of three independent experiments performed in duplicate. Error bars represent standard deviation. Statistical analysis was performed using one-way ANOVA with Dunnett’s multiple comparisons test (*,P ≤ 0.05; **** P ≤ 0.0001; ns, non-significant).

Some viruses are able to use multiple receptors on different cell types. Thus, we set out to investigate whether LGTV attachment to the different NCI60 cells could be mediated by different proteins. Plasma membrane peptides were extracted by trypsinization from two cell lines shown to have high LGTV binding (A549 and SW620) and were used for pre-incubation with LGTV before binding. Membrane protein extract from OVCAR5, one of the NCI60 cell lines with little to no LGTV binding, was used as negative control. The viability of the cells after pre-incubation with the tripsinized membrane protein extracts was confirmed by trypan blue exclusion test [38]. When LGTV was incubated with 100 and 10 μg/mL of membrane trypsinized peptide extract from A549, no decrease in binding to either A549 or SW620 was observed (Fig 9A). However, when 100 μg/mL of the membrane trypsinized peptide extract from SW620 cell line was used, we observed a decrease in binding to SW620 of 50% but not to A549 cell line (Fig 9B). Furthermore, the pre-incubation of LGTV with the trypsinized membrane extract from the control cell line OVCAR5 did not induce any statistically significant decrease in binding to the two cell lines tested (Fig 9A and 9B). These results suggested that the attachment receptor(s) for LGTV present on A549 cells might be different from those present on SW620 cells.

## Discussion

Virus-host interactions such as attachment and entry into host cells are essential first steps of the virus life-cycle. A better knowledge of these interactions is crucial in order to better understand virus tropism and pathogenesis. In simple situations, for a virus to enter into a host cell, it attaches to its specific receptor with high affinity, which leads to virus uptake and entry. However, this process can be far more complex, involving the cooperation of several molecules for attachment and entry, either used simultaneously, sequentially or in a cell-type-specific manner.

Some viruses, including TBEV, have been suggested to bind to attachment receptors in a relatively nonspecific manner through reversible associations with the cell membrane via generalized biochemical properties, such as charge. However, several flaviviruses, have been shown to bind specifically to sophisticated patterns to attach to receptors, which act as both attachment and entry receptors [39–41].

Furthermore, virus-cell attachment has a high potential for prophylactic or therapeutic ends, yet only a few drugs have been licensed to date that target this crucial first stage in the virus life cycle [42].

For several flaviviruses, multiple receptors have been suggested, however, in most studies no difference has been made between attachment and entry receptors. This is the case for TBEV, for which interaction with three receptors has been suggested glycosaminoglycan heparan sulfate (HS) [15], human laminin*-*binding protein (LBP) [16] and human integrin αVβ3 [17]), yet so far, no functional studies have been done in order to demonstrate that these are entry receptors, leading to the assumption that the main receptor(s) remain to be identified.

In this study, LGTV was used as a model for TBEV since it is the naturally attenuated member of the TBEV complex, it is closely related to TBEV, sharing many protective E protein epitopes as well as 82–88% of amino acid identity [8]. LGTV has been successfully tested as a live-attenuated vaccine for TBEV. Unfortunately, this vaccine was discontinued due to the occurrence of encephalitis in 1:10000 of vaccine recipients[43]. Yet, under natural conditions it is not associated with human disease, making it a good model to study TBEV.

Here, we employed two steps to generate a list of cellular attachment/entry receptor candidates for LGTV. In the first step we characterized the biochemical nature of the LGTV-host attachment receptor, as virus receptors can be of protein, glycoprotein, proteoglycan, or glycolipid nature. Based on this, we developed a transcriptomic-based approach which led to a number of candidate receptors.

To characterize the biochemical nature of the host cellular attachment receptor(s) for LGTV, we made use of several compounds that either metabolically inhibit or enzymatically cleave glycolipids, proteins, and glycans on the cell surface. According to our results, LGTV binding to A549 cells was shown to be independent from the presence of glycolipids. This contrasts with what was described for the flavivirus Dengue virus (DENV), for which glycosphingolipids were shown to be potential attachment factors in both mosquito and mammalian cells [44]. Moreover, plasma membrane proteins were shown to have a role in the attachment of LGTV to A549 cells, as demonstrated by the decrease of virus binding after cleavage of cell surface proteins using the proteases Ficin and Proteinase K (Fig 1). This suggested that LGTV was either able to bind directly to one or more proteins on the cell surface or to a glycan component present on these proteins. To clarify this, we performed binding assays after treatment of cells with compounds that inhibit or cleave different glycan types that can be found on cell surface proteins, *viz*. HS, *N*-glycans, *O*-glycans and SA. We also made use of CHO cells expressing or lacking *N*-glycans and SA. Our results suggested that none of the glycans tested are components of the cellular attachment receptor for LGTV. Of interest, we found that LGTV attachment to A549 cells was not dependent on the presence of HS (Fig 2). This contrasts with what was described for other flaviviruses [45–49], including TBEV, for which HS has been shown to be a major determinant of attachment [15]. Despite the fact that the affinity for HS has been shown to be a factor of virus attenuation *in vivo* [48], for LGTV, the naturally attenuated TBEV, this was not observed. Moreover, Mandl *et al*. suggested the affinity of TBEV to HS to be the consequence of cell culture adaptation, when BHK-21 cells were used to amplify the virus [50].

According to our results, LGTV binding to host cells was shown not to be *N-*glycan dependent. Rather, a statistically significant increase in binding was observed both when *N*-glycans were removed or absent. Furthermore, a similar pattern was observed in the absence of SA. The cell surface of mammalian cells is homogeneously covered with high amounts of glycans, the glycocalyx, which is the first physical structure encountered as the cell surface is approached [51]. Compared to their protein backbones, which are prone to folding, glycan chains tend to occupy much more space per mass thus, it is likely that the removal of *N*-linked glycans and/or SA, increases the surface exposed of the attachment receptor protein.

Taken together, these results led us to conclude that the cellular attachment receptor for LGTV is most likely of protein nature. This is in agreement with what has been previously suggested for TBEV and other flaviviruses. Indeed, multiple proteins have been suggested as receptors for different flaviviruses, including TBEV [16,17], DENV, West Nile virus and Japanese encephalitis virus (reviewed elsewhere [14,52]).

Based on the assumption that the cellular attachment receptor for LGTV is of protein nature, we made use of the NCI60 panel of cell lines to generate a list of candidate receptors by correlating the level of LGTV binding with the mRNA expression levels of each NCI60 cell line. The results showed that LGTV is able to bind to cells of lung, colon, CNS, skin, ovary, kidney, and breast origin (Fig 7). The broad distribution of binding of LGTV to most organs and cell types tested, indicates that either the attachment factor(s) are ubiquitously expressed in these different cell types/organs, or that LGTV is able to attach to different receptors in different cell types.

For TBEV, dendritic cells (DCs) in the epidermis have been shown to be early target cells [53,54], followed by the lymph nodes, where it replicates and further spreads to secondary visceral organs and eventually crosses the blood-brain barrier into the CNS. The binding results shown here, lead us to speculate that the tropism might not be defined solely by the distribution of the attachment receptors but rather to other cell-dependent factors. Indeed, *in vivo* studies have shown the interferon system to be a determinant factor in LGTV tropism in mice. When the interferon pathway was abrogated, LGTV could be detected in all peripheral organs, spinal cord, and brain, while in wild type mice, virus could only be transiently detected in the brain [55,56]. Furthermore, LGTV and TBEV are known to be neurotropic viruses that cause encephalitis and have been shown to infect neurons and astrocytes [56–59].

Contrarily to what was expected, LGTV did not show high binding to CNS cell lines (17 to 48% as compared to the A549 control). Altogether, these results lead to the assumption that the attachment factor(s) for LGTV might not be highly expressed on the target cells surface, and that the tropism might be the consequence of the presence of the attachment receptor(s) combined with the biology of the virus within the host cells.

Interestingly, our results suggested a variable binding pattern within a same cell type/organ. A possible explanation for this, could be the fact that immortalization and adaptation of cells to culture can lead to genotypic and phenotypic drifting, which involves both the expression of proteins not normally expressed, as well as the down-regulation of proteins normally expressed. Cell lines can lose many of the specific functions typical of the tissue and cell type *in vivo*, since it shifts more of its resources into functions associated with proliferation [60].

We hypothesized that LGTV was able to use different attachment receptors in different cell types. To test our hypothesis, we enzymatically cleaved the membrane proteins from the cell lines A549 and SW620 (cell lines for which the highest LGTV binding was observed among the NCI60 panel) and used these peptide extracts to pre-incubate with LGTV in an attempt to block binding. We observed that SW620 membrane peptides were able to decrease LGTV binding to SW620 cells but not to A549, whereas A549 membrane peptides were not able to block binding to any of the two cell lines (Fig 9). This leads to the speculation that LGTV could be able to bind to different receptors in different cell types. In fact, some viruses are able to use different receptors depending on the target cell. For example, for DENV, different receptors have been suggested on different cell types: DC-SIGN or L-SIGN on DCs [61,62]; LPS-CD14 complex on macrophages and monocytes [63]; the mannose receptor on macrophages [64]; and GRP 78 [65,66] and laminin receptor [67] on hepatocytes. These findings are of importance for future studies, particularly when designing experiments to test the role of specific candidates in the entry of LGTV and/or TBEV. The possibility of having cell-type specific receptors should be taken into account when choosing the strategy and the cell models to be used.

From the candidate receptor list generated, several proteins were found to be related to the Wnt pathway (LGR6 [68], NKD1 [69], FZD10 [70] and SYT1 [71]). Others were found to be expressed predominantly in the CNS (GPR155 [72], KCNK10 [73] and RIMBP2 [74]), or the periferic nervous system (PNS) (GFRA3 [75]); or to have a role in the maturation of DCs (TMEM176A and TMEM176A [76]), among others. This candidate receptor list has the limitation of being based on the transcription levels of genes, which does not always correlate with the expression level of proteins. Nonetheless, our results provide a list of genes with significant mRNA expression in cell lines with high LGTV binding.

From the selected receptor candidates, LGR6 was the one with the highest correlation value. LGR6 is one of the receptors for R-spondins, which are potent Wnt signal enhancers [68] and was shown to be a marker of stem cells in the hair follicle, which is responsible for generating all cell lineages of the skin [77]. Also, LGR6 was shown to localize with the surrounding nerve endings and their corresponding Schwann cells [78]. Our results indicate that LGR6 is most likely not a receptor for LGTV in A549 and SW620, however, these results do not exclude the possibility of LGR6 being a receptor for LGTV in other cell types. Further studies are needed to investigate the role of LGR6 and other Wnt pathway related proteins in binding and entry of LGTV and TBEV into host cells. Of note, it should be taken into account that the cell line with the highest LGTV binding (SW620) is a colon cancer cell line, known to have aberrant Wnt pathway [79], indicating that the Wnt related candidates might be decoy results and that the specific receptor(s) might be among the other candidates.

In conclusion, we present here evidence that LGTV cellular attachment receptor(s) are most likely of protein nature and we suggest that LGTV-host interactions might involve more than one single host cell-surface molecule in a cell-type dependent manner. Furthermore, we propose a list of selected attachment/entry receptor candidates which might serve as a basis for future receptor studies for LGTV and TBEV.

## Materials and methods

### Cells and media

The human lung carcinoma cell line A549 (gift from Dr. Alistair Kidd) and the human embryonic kidney (Hek) 293 cell line (provided by Dr. Friedemann Weber) were maintained in Dulbecco’s modified Eagle medium (DMEM) supplemented with 5% foetal bovine serum (FBS) (HyClone), 20 U/ml penicillin and 20 μg/ml streptomycin (Invitrogen). Chinese hamster ovary (CHO) cell lines Pro-5, Lec1 and Lec2 (both purchased from LGC Promochem) were maintained in minimal essential medium (MEM) supplemented with 5% FBS, 20 U/ml penicillin and 20 μg/ml streptomycin. The NCI-60 panel of human tumor cell lines was obtained from the Developmental Therapeutics Program (DTP; National Cancer Institute, NIH). These cell lines were cultured as directed by the DTP, using RPMI 1640 (Invitrogen) supplemented with 10% FBS, 20 U/ml penicillin and 20 μg/ml streptomycin and 1 mM L-glutamine (Hyclone). All confluent cells were split the day before an experiment.

### Virus production and purification

Confluent A549 cells were incubated with LGTV at an MOI of 0.01 for 1 h at 37°C with 5% CO_2_. The inoculum was discarded and replaced with fresh medium, and cells were incubated for 72 h. The virus-containing supernatant was cleared at 3100×g for 20 min at 4°C. All subsequent centrifugations were done using the Optima L-80 XP ultracentrifuge (Beckman Coulter). Purification of supernatant containing virus was done by ultracentrifugation through a sucrose gradient of 20% and a 60% sucrose solutions in TN buffer (0.1 M NaCl, 0.05 M Tris-HCl pH 7.4) at 140 000×g for 90 min at 4°C in a SW 32 rotor. The layer between 20% and 60% sucrose was harvested and the sucrose concentration was adjusted to 55%. Flotation centrifugation was performed by adding 30% and 10% sucrose solutions in TN buffer at 150 000×g for 16 h at 4°C in a SW 32 rotor. The layer underneath the 30% sucrose layer was harvested and centrifuged at 140 000×g in a SW 41 rotor for 90 min at 4°C. The resulting pellet was resuspended in binding buffer (BB: DMEM, 20 mM Hepes, 20 U/ml penicillin + 20 μg/ml streptomycin, 1% BSA) or PBS and stored at -80°C. The purity of the concentrated virus was determined by SDS-PAGE and Western blot, and the titre (in focus forming units, FFU) was determined by focus forming assay as described by Överby et al. [80].

### Binding experiments

Cells were detached using PBS with 0.05% EDTA and reactivated in growth medium for one hour at 37°C (in solution), pelleted in 96 well plates (1.5×10^5^ cells/well) and washed with BB. In order to have 70% of cells with attached virus, LGTV was added at an MOI of ~10 in BB (titrated in the A549 cell line) and the cells were incubated for 1 h at 4°C on ice with agitation. Unbound virions were washed away with PFN buffer (PBS, 2% FCS, 0.01% NaN3) and the cells were subsequently incubated with primary antibody (mouse anti-E mAb 1786 [81]) 1:500 in PFN and secondary antibody (donkey anti-mouse IgG (H+L), Alexa Fluor 488 conjugated (Life Technologies)) 1:1000 in PFN, both for 30 min at 4°C on ice with agitation. The amount of virus bound to cells was measured using BD LSR II Flow Cytometer (BD Biosciences) and results were analyzed with software BD FACSDiva 8.0 (BD Biosciences). Results from LGTV binding experiments were acquired as geometrical mean. The following additions/variations of binding experiments were performed:

1. After reactivation, cells were treated either without (untreated control) or with increasing amounts of the following proteases: 10 and 100mU/mL of the sulfhydryl protease Ficin from fig tree latex (Sigma) (one unit produces a ΔA280 of 1.0 per minute at pH 7.0 at 37 °C when measuring TCA soluble products from casein in a final volume of 10 ml (1 cm light path)) [18]; and 100mU/mL and 1U/mL of the serine protease Proteinase K from *Engyodontium album*. (Sigma) (one unit hydrolyzes urea-denatured hemoglobin to produce color equivalent to 1.0 μmole of tyrosine per min at pH 7.5 at 37 °C (color by Folin-Ciocalteu reagent)) in PBS for 1h at 37°C with agitation. Subsequently, the cells were washed thrice with PBS on ice in order to remove all traces of protease prior to the addition of the virus. Cell viability was verified using the trypan blue exclusion assay, as described elsewhere [38]. As a control, cells were incubated with 10 000 vp/cell of 35S-labelled virions of HAdV-37, known to bind to proteins on the cell surface [21,22], for 1 h on ice at 4°C with agitation. The associated radioactivity was measured in a Wallac 1409 liquid scintillation counter (Perkin-Elmer). The results were acquired as counts per minute (CPM).
2. To enzymatically remove HS, after reactivation, A549 cells were treated either without (untreated control) or with increasing amounts of Heparinase III from Flavobacterium heparinum (Sigma) (one unit forms 0.1 μmole of unsaturated uronic acid per hr at pH 7.5 at 25 °C) [23] in PBS for 1h at 37°C with agitation. Subsequently, the cells were washed thrice with PBS on ice in order to eliminate Heparinase III prior to the addition of the virus. Cell viability was verified using the trypan blue exclusion test, as described elsewhere [38]. As a control, cells were incubated with 10 000 vp/cell of 35S-labelled virions of HAdV-5 pre-incubated with FX, known to bind to HS [82–84] for 1 h on ice at 4°C with agitation. The associated radioactivity was measured as described above.
3. To enzymatically remove N-linked glycans, after reactivation, the cells were treated either without (untreated control) or with 100 mU/mL PNGase F from *Elizabethkingia meningoseptica* (Sigma) (one unit catalyzes the release of N-linked oligosaccharides from 1 nanomole of denatured ribonuclease B in one minute at 37°C at pH 7.5) [25] in PBS for 1h at 37°C with agitation. Subsequently, the cells were washed thrice with PBS on ice in order to remove all PNGase prior to the addition of the virus. Cell viability was verified using the trypan blue exclusion test, as described elsewhere [38]. As controls for the removal of *N-*linked glycans, cells were incubated with fluorescein-labelled *Phaseolus Vulgaris Erythroagglutinin* (E-PHA) (Vector Laboratories), at the concentrations of 0.3 μg/ml for A549 cells and 3 μg/ml for Pro-5 and Lec1 cells in E-PHA buffer (10 mM Hepes, 0.15 M NaCl) for 1 h on ice at 4°C with agitation.
4. Cells were pre-treated either without (untreated control) or with 3 mM of the *O*-glycosilation synthesis inhibitor Benzyl-α-GalNAc [27] for 48 h at 37°C, and also during the 1 h reactivation period prior to binding with LGTV. Subsequently, the cells were washed thrice with PBS on ice in order to remove Benzyl-α-GalNAc prior to the addition of the virus. Cell viability was verified using the trypan blue exclusion test, as described elsewhere [38]. As a control for the removal of *O*-linked glycans, cells were incubated with 10 000 vp/cell of ^35^S-labelled virions of HAdV-37, shown to bind to *O*-linked GD1a glycans on glycoproteins [22], for 1 h on ice at 4°C with agitation. After a washing step, the associated radioactivity was measured as described above.
5. Cells were pre-treated either without (untreated control) or with 2.5 μM of the glycolipid synthase inhibitor P4 (DL-threo-1-phenyl-2-palmitoylamino-3-pyrrolidino-1-propanol), in its active (1*R*,2*R*) or inactive (1*S*,2*S*) form [28], for 5 days at 37°C, and also during the 1 h reactivation period prior to binding with LGTV. Subsequently, the cells were washed thrice with PBS on ice in order to remove all traces of P4 prior to the addition of the virus. Cell viability was verified using the trypan blue exclusion test, as described elsewhere [38]. As a control for the effect of P4, cells were incubated with 10 μg/ml ganglioside GM1-binding FITC-conjugated CT-B diluted in PBS-0.1%BSA for 1 h on ice at 4°C with agitation.
6. To enzymatically remove sialic acid monosaccharides, after reactivation, the cells were treated either without (untreated control) or with 10 mU/mL of neuraminidase from *Vibrio Cholerae* (Sigma), an exosialidase which hydrolyzes α-ketosidic linkage between the sialic acid and an adjacent sugar residue hich hydrolyzes (one unit hydrolyzes 1 μmol N-acetyl-neuraminosyl-D-lactose within 1 min at +37 °C) [31] in PBS for 1h at 37°C with agitation. Subsequently, the cells were washed thrice with PBS on ice in order to remove all traces of neuraminidase prior to the addition of the virus. Cell viability was verified using the trypan blue exclusion assay, as described elsewhere [38]. As a control for the removal of the SA, cells were incubated with 10 000 vp/cell of 35S-labelled virions of HAdV-37, known to bind to sialic acids on glycoproteins [32], for 1 h on ice at 4°C with agitation and the radioactivity was quantified as described above.
7. After reactivation, A549 and SW620 cells were pre-incubated either without (untreated control) or with 10-fold dilutions (1/100, 1/1000 and 1/10000) of two rabbit pAbs α-LGR6: α-LGR6 LL (Proteintech) raised against peptide 1 to 351 and α-LGR6 HR (Novus Biologicals) raised against peptide 421 to 565, for 1 h on ice at 4°C with agitation. Subsequently, the cells were washed thrice with PBS on ice in order to remove the antibodies prior to the addition of the virus. Cell viability was verified using the trypan blue exclusion test, as described elsewhere [38].
8. Before binding to reactivated A549 and SW620 cells, LGTV was pre-incubated either without (untreated control) or with 50 and 5 μg/mL of recombinant human LGR6, 25 to 567aa, (Novus Biologicals) in cold PBS, for 1 h on ice at 4°C with agitation. Cell viability was verified using the trypan blue exclusion test, as described elsewhere [38].

### Correlation of LGTV binding with microarray gene expression pattern

The NCI60 cancer cell line panel is a list of 60 well characterized cell lines, for which the gene transcript expression levels was determined and data is publicly available as part of the Molecular Target Program (https://dtp.cancer.gov/databases_tools/molecular_target/default.htm). The RNA expression levels included in these databases were determined using four microarray platforms [33–37].

Binding experiments were performed in groups of 8 to 16 NCI60 cell lines at once, using the same protocol as described above. The flow cytometer settings were kept constant during the complete experimental procedure. In order to assess inter-experimental consistency and to provide a positive control, A549 cells were used to bind in parallel with the NCI60 cells in each experiment. The binding results obtained were normalized to the positive controls using the following formula: (Geometrical Mean of NCI60 cell line incubated with LGTV—Geo Mean of NCI60 cell incubated with buffer) / (Geo Mean of A549 incubated with LGTV—Geo Mean of A549 incubated with buffer)*100. Cell lines with inconsistent results among the replicates were excluded from the analysis.

Analysis with Pearson correlation coefficients was performed in order to compare the binding values obtained for LGTV to the Molecular Targets RNA database (13695 comparisons). This analysis generated a list of transcripts ordered from the highest correlation values to the lowest. Selection of relevant candidates from this extensive list was performed based on the highest correlation values and p value <.0001. Proteins corresponding to the selected RNA transcripts were searched in Uniprot (http://www.uniprot.org/), a database of protein sequences and functional information. Those shown not to be cell surface-associated were excluded from the candidate list.

### Inhibition of LGTV binding using membrane trypsinized peptide extracts

A549, SW620 and Ovcar5 cell lines, cultured in 175 cm^2^ flasks, were washed with PBS and subsequently incubated for 5 min at 37°C with 800 μL of 0.05% Trypsin-EDTA (GIBCO). The cells were pelleted and the supernatant containing the trypsinized membrane peptide extract was diluted to the concentrations of 100 and 10 μg of peptides/mL in BB, which were used to pre-incubate with LGTV for 1h at 4°C, prior to binding to A549 and SW620 cell lines. The controls were treated as follows: LGTV was pre-incubated in BB with the same volume of Trypsin-EDTA used to obtain the concentration of 100 μg of peptides/mL, to ensure that the residual amount of Trypsin-EDTA has no detrimental effect on the viral surface proteins. Binding was performed as described above.

### Overexpression of LGR6 in Hek293 cells

Plasmid pIRESpuro3, containing the human LGR6 ORF, fused with the CD8 signal peptide and flag-tag at the N-terminus, was kindly provided by Dr. Qingyun Liu. Hek293 cells were transfected using GeneJuice (Merck Millipore) and selected with puromycin at 1 μg/mL. The overexpression of flag-LGR6 was confirmed by flow cytometry and western blotting. Briefly, Hek293 and Hek293-flag-LGR6 cells were detached with PBS-EDTA, reactivated in growth medium for 1h at 37°C, pelleted in V-shaped 96 well plates (1.5x10^5^ cells/well) and washed with PBS. The cells were subsequently incubated with primary antibody (mouse mAb directed against flag-tag (M2, Stratagene)) 1:1000 in PBS and secondary antibody (donkey anti-mouse IgG (H+L), Alexa Fluor 488 conjugated) 1:1000 in PBS, both for 30 min at 4°C on ice with agitation, followed by a washing step before flow cytometry analysis.

For western blotting, the cell lysates from Hek293 and Hek293-flag-LGR6 cells were resolved on 10% Bis-Tris denaturing gels (NuPAGE, Invitrogen, Life Technologies) and transferred to Trans-Blot nitrocellulose membrane (Bio-Rad Laboratories, Solna, Sweden) by electroblotting. After a blocking with 5% milk in PBS-T (PBS supplemented with 0.05% Tween20) for 1h at RT, staining was carried out using 1:5000 of the anti-flag-tag mAbs, followed by a 1:1000 dilution of a HRP-conjugated rabbit anti-mouse IgG antibody (Dako), both in PBS-T with 2.5% milk. The flag-tag was then detected by chemiluminescence using super signal west femto (Thermo Scientific) and visualized using the multipurpose CCD camera system FujiFilm LAS-4000.

## Supporting information

S1 Table Candidate receptor list. The binding pattern of LGTV to 50 of the 60 cell lines of the NCI60 panel of cancer cell lines was compared to the known gene expression patterns of each cell line. The candidate receptors highlighted were selected based on the correlation value, p value and presence on the plasma membrane.
(XLSX)
